# Genetic Association between Methylenetetrahydrofolate Reductase Gene Polymorphism and Risk of Osteonecrosis of the Femoral Head

**DOI:** 10.1155/2015/196495

**Published:** 2015-01-26

**Authors:** Wei Chai, Zhendong Zhang, Ming Ni, Peiliang Geng, Zijian Lian, Guoqiang Zhang, Lewis L. Shi, Jiying Chen

**Affiliations:** ^1^Department of Orthopaedics, Chinese People's Liberation Army General Hospital, 301 Orthopaedic Hospital, 28 Fuxing Road, Haidian District, Beijing 100853, China; ^2^Cancer Center, Division of Internal Medicine, Chinese PLA General Hospital & Chinese PLA Medical School, 28 Fuxing Road, Haidian District, Beijing 100853, China; ^3^Medical School of Nankai University, 94 Weijin Road, Tianjin 300071, China; ^4^Department of Orthopaedics, University of Chicago Hospital, 5841 S. Maryland Avenue, Chicago, IL 60637, USA

## Abstract

*Background*. Methylenetetrahydrofolate reductase (MTHFR) SNP rs1801133 has been frequently investigated in recent years. Relevant candidate gene association studies with this SNP and osteonecrosis of the femoral head (ONFH) reported conflicting results. Meta-analysis provides a method to combine these data and to determine the association in a larger sample size. *Method*. We conducted a systematic search to identify possible studies. Four pooled ORs (odds ratios, T versus C, TT versus CC, TT/CT versus CC, and TT versus CT/CC), along with 95% confidence interval (CI), were calculated to evaluate the association between SNP rs1801133 and ONFH susceptibility. Both fixed effects model and random effects model were used. *Findings*. We eventually included twelve studies in this analysis, with results showing no overall association between ONFH susceptibility and SNP rs1801133 (T versus C: OR = 1.15, 95% CI = 0.97–1.38; TT versus CC: OR = 1.15, 95% CI = 0.91–1.46; TT/CT versus CC: OR = 1.09, 95% CI = 0.95–1.25; and TT versus CT/CC: OR = 1.16, 95% CI = 0.93–1.45). When stratified based on ethnicity, the results were still not significant. *Conclusion*. Our findings are generally supportive of no association between MTHFR SNP rs1801133 and the etiology of ONFH.

## 1. Introduction

Osteonecrosis of the femoral head (ONFH) is a painful disorder of the hip and features necrosis of femoral subchondral bone [1]. This disease is often observed in young adults and is frequently progressive. ONFH will often evolve into subchondral bone fracture, femoral head collapse, and subsequent hip joint degeneration, leading to poor long term outcomes [2]. The collapse of femoral head often leads to loss of function [3–5]. The pathophysiology of ONFH remains incompletely elucidated; intravascular coagulation may represent a pathogenetic mechanism resulting in bone ischemia and death [6]. Coagulation abnormalities in patients with hip osteonecrosis are associated with an increase in risk of developing bone necrosis by predisposing thromboembolic phenomena [7].

A large number of outstanding researchers have identified multiple factors that genetically contribute to hypercoagulability and osteonecrosis, including protein C and protein S deficiency, factor V Leiden, prothrombin G20210A mutation, and higher lipoprotein levels [8–10]. In recent studies, several gene single nucleotide polymorphisms (SNPs) associated with thrombophilia have been implicated in ONFH [11]. Methylenetetrahydrofolate reductase (MTHFR) is an enzyme which plays a key role in the remethylation of homocysteine. A C→T mutation at nucleotide 677 leading to a valine substitution for an alanine (677C > T, rs1801133) is reported to be functionally relevant to MTHFR enzymatic activation, homocysteine levels, and thrombotic events [12, 13].

Extensive research into the etiology of ONFH to date has focused on SNP rs1801133. Results of a study by Glueck et al. in 2001 showed that SNP rs1801133 was a genetic risk factor predisposing susceptibility to ONFH [14]. Recently, Li et al. investigated the association of the SNP with risk of ONFH in a sample of Chinese patients, finding supportive evidence of a significant association as well [15]. However, most replication studies reported that SNP rs1801133 is not a risk factor for the bone disease [16–18]. Hence, we hypothesize that the ONFH susceptibility risks may not be attributable to SNP rs1801133. In an effort to test this hypothesis, we performed a meta-analysis to combine all published data and to determine the association in a larger sample size.

## 2. Methods

### 2.1. Study Search and Selection

A systematic search of the PubMed (http://www.ncbi.nlm.nih.gov/sites/entrez/) and CNKI (http://www.cnki.net/) electronic database was conducted up to October 1, 2013, to identify all studies investigating the association between SNP rs1801133 and ONFH risk. “Osteonecrosis of the femoral head,” “ONFH,” “Methylenetetrahydrofolate reductase,” “MTHFR,” and “polymorphism” were the keywords we used in the search. The electronic search was complemented with a manual search of citations of all original articles.

There were three inclusion criteria for the meta-analysis. First, the study must investigate the association between SNP rs1801133 and ONFH risk by comparing ONFH patients with healthy or ONFH-free control subjects. Second, the study had to provide detailed genotype frequency or contain sufficient data with which we could calculate the frequencies of TT, CT, and TT. Third, the subjects must be humans and cannot be repeatedly used; if they were, we included the study with the largest number of subjects.

### 2.2. Data Extraction

Using the inclusion criteria listed above, two investigators picked out all eligible studies and extracted the following data from each of them: authors, year of publication, country of study, ethnicity, genotyping method, genotypic frequencies, and type of controls. Disagreements were resolved by discussion.

### 2.3. Statistical Analysis

We first checked the deviation from Hardy-Weinberg equilibrium (HWE) in control subjects using a chi-squared goodness-of-fit test. To evaluate the strength of the genetic association, four pooled ORs (odds ratios, T versus C, TT versus CC, TT/CT versus CC, and TT versus CT/CC) were calculated, along with the 95% confidence intervals (CIs). A statistical test described by Cochran was conducted to measure heterogeneity between studies [19]. In case of absence of heterogeneity, the pooled ORs were calculated assuming a fixed effects model [20], or a random effects model was used [21]. To identify if included studies influenced summary estimates substantially, we performed sensitivity analysis by omitting one study at a time. Publication bias was qualitatively assessed using the funnel plots and Egger's test [22]. All statistical tests were performed with the software Stata version 12.0 (Stata Corporation, College station, TX, USA). *P* value <0.05 was considered as significant.

## 3. Results

### 3.1. Extraction Process and Study Characteristics

**Figure 1:**
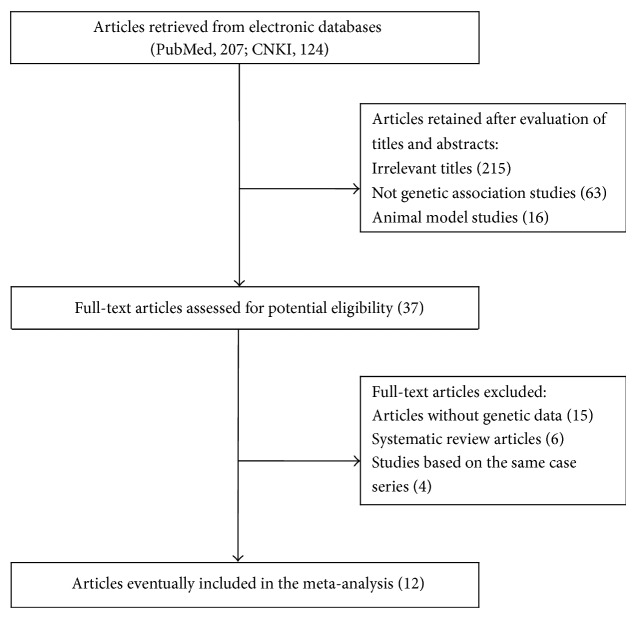
Flow chart of study identification and specific reasons for exclusion from the meta-analysis.

Figure 1 summarizes the process of study identification. We yielded a total of 331 relevant articles (PubMed, 207; CNKI, 124). Evaluation of title, abstract, and full-text led to 12 eligible studies [14–18, 23–29]. General characteristics of the included studies are summarized in Table 1. Seven studies investigated the association of SNP rs1801133 with ONFH susceptibility in a sample of Caucasian population and five in Asian populations. Seven studies used healthy control subjects and four used ONFH-free subjects, with one not reporting such information. Most studies used polymerase chain reaction-restriction fragment length polymorphism (PCR-RFLP) to genotype SNP rs1801133. Deviation from HWE was tested in three studies [15, 16, 26].

### 3.2. Meta-Analysis Results

**Figure 2:**
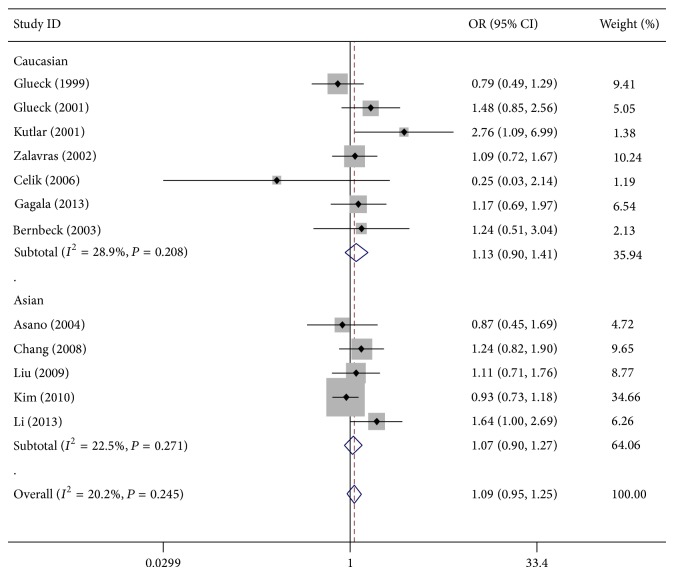
Meta-analysis using a fixed effects model for the association between MTHFR SNP rs1801133 and ONFH susceptibility (TT/CT versus CC) is illustrated in analysis stratified by ethnicity. OR: odds ratio; CI: confidence interval.

As shown in Table 2, all of the genetic models we tested showed that individuals with T allele appeared to be at higher risk of ONFH (T versus C: OR = 1.15, 95% CI = 0.97–1.38; TT versus CC: OR = 1.15, 95% CI = 0.91–1.46; TT/CT versus CC: OR = 1.09, 95% CI = 0.95–1.25; and TT versus CT/CC: OR = 1.16, 95% CI = 0.93–1.45) (Figure 2), although this was not statistically significant. In terms of stratified analysis by ethnicity, the results for both Caucasian and Asian populations were similar to those for the overall population (Table 2 and Figure 2).

### 3.3. Heterogeneity Test and Sensitivity Analyses

We observed no significant heterogeneity for SNP rs1801133 under all genetic models with the exception of T versus C (*P*_Heterogeneity_ = 0.027) (Table 2). Sensitivity analyses were conducted, and it was identified that Li et al. [15] and Kutlar et al. [16] were the main sources of heterogeneity, exclusion of which effectively increased homogeneity without significantly influencing the estimates of overall ONFH risk (*P*_Heterogeneity_ = 0.223, OR = 1.03, 95% CI = 0.92–1.17). This outcome assured the stability and reliability of our results.

### 3.4. Publication Bias

**Figure 3:**
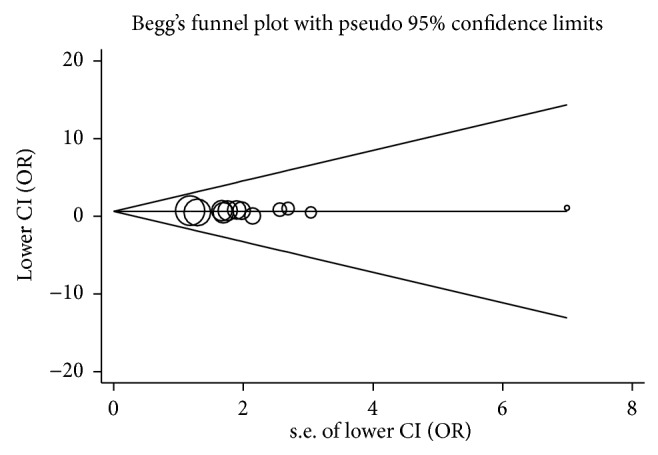
Funnel plot of publication bias for TT/CT versus CC. Begg's test *P* = 0.653; the circles represent the weight of each study.

We evaluated the publication bias using the tests described by Begg and Egger. The shape of all funnel plots was symmetrical (*P* > 0.05). Begg's test also showed that there was little publication bias across studies (*P* > 0.05) (Figure 3 for TT/CT versus CC).

## 4. Discussion

SNPs represent the most common type of DNA variation in human. Genetic association studies are being widely and appropriately used to identify potential genetic prognostic factors and molecular biomarkers for genetically determined human diseases [30]. Due to their impact on the biological function of corresponding genes, candidate gene association studies have given priority to the well-characterized SNPs such as rs1801133 in exon 4 of the MTHFR gene. Kim et al. conducted a comparison of ONFH and control subjects using logistic regression models, revealing no significant association between SNP rs1801133 and ONFH susceptibility in a Korean population [28]. Chang et al., however, showed evidence that supported the involvement of this SNP in development of ONFH [26]. The disparate results across the studies may at least partly be attributable to the relatively small sample sizes. This led us to perform a meta-analysis to determine the association in a larger sample size.

When pooling all published data together, we found little evidence of a significant association between SNP rs1801133 and ONFH susceptibility, consistent with our hypothesis. A review of overall progress and contribution of candidate gene association studies to current understanding of the genetic susceptibility to cancer suggested that MTHFR C677T increased risk of gastric cancer (OR, 1.52; 95% CI, 1.31–1.77) [31]. Variation of SNP rs1801133 is associated with decreased enzymatic activity and elevated homocysteine levels. Increased frequency of this SNP has been detected among patients with cardiovascular disease and coronary atherosclerotic disease [12, 32]. These data are suggestive of a possible effect on human diseases attributed to SNP rs1801133. On the other hand, we cannot exclude the possibility that these diseases are etiologically different and it is likely that SNP rs1801133 may not represent a contributing factor for ONFH. However, this aspect is worth to be proven with a very large-sized study.

Further analysis stratified by ethnicity also showed no association in Asians and in Caucasians. It has previously been reported that CT and TT genotypes have 65% and 30% of the MTHFR activity, respectively, suggesting individuals with these genotypes are at higher risk of various diseases, such as ONFH [33]. A frequency ranging from 5% to 40% in European populations [32] implicated that SNP rs1801133 may contribute to ONFH, especially among Caucasians. But the expected association was not detected in our study, which may still be caused by the limited sample size.

Results of a previous meta-analysis showed an association between MTHFR SNP rs1801133 and ONFH in non-Asian populations [34]. In this analysis, a total of eight studies (five on Caucasians) with 778 cases and 1,162 controls were included, possibly leading to a less reliable and incomprehensive conclusion because of the smaller sample. We expanded our analysis by adding additional four studies, with no significant association identified. The uncertainty highlights the necessity to conduct subsequent studies to validate the presently unclear genetic association.

There are some limitations to this study. The ONFH individuals in our study were caused by different etiologies, including primary ONFH without special history, glucocorticoid intake, and alcohol abuse, which may affect the final results. There were significant heterogeneity among the studies, including ethnicity, number of individuals, and methodology. The total sample size may not generate sufficient power to detect the overall and ethnicity-specific effects in subgroups, and minor or moderate association may be masked. Additionally, the nonstandardized selection of control population, such as healthy versus ONFH-free subjects, may introduce nondifferential misclassification bias. Lastly, estimation of age in ONFH susceptibility was not conducted due to lack of original data.

In conclusion, the current meta-analysis revealed that MTHFR SNP rs1801133 was not significantly associated with the development of ONFH. We found similar results in ethnicity-specific analysis. Considering ONFH can be caused by many factors such as application of glucocorticoid and alcoholism, these findings need further validation through future larger studies stratified by different etiological factors.

## Figures and Tables

**Table 1:** General characteristics of the studies included in the present meta-analysis.

ID	Study	Publication year	Ethnicity (country)	Control source	Genotyping methods	Sample size	
						Case	Control
1	Glueck et al. [23]	1999	Caucasian (USA)	Healthy subjects	PCR-RFLP	59	234
2	Glueck et al. [14]	2001	Caucasian (USA)	Healthy subjects	PCR-RFLP	36	235
3	Kutlar et al. [16]	2001	Caucasian (USA)	ONFH-free subjects	PCR-RFLP	45	62
4	Zalavras et al. [24]	2002	Caucasian (Greece)	Healthy subjects	PCR-RFLP	66	300
5	Bernbeck et al. [17]	2003	Caucasian (Germany)	NS	NS	14	73
6	Asano et al. [18]	2004	Asian (Japan)	ONFH-free subjects	DS	31	106
7	Celik et al. [25]	2006	Caucasian (Turkey)	ONFH-free subjects	PCR-RFLP	11	39
8	Chang et al. [26]	2008	Asian (Korea)	Healthy subjects	Multiplex PCR	71	200
9	Liu et al. [27]	2009	Asian (China)	Healthy subjects	PCR-RFLP	89	77
10	Kim et al. [28]	2010	Asian (Korea)	ONFH-free subjects	TaqMan	438	269
11	Gagala et al. [29]	2013	Caucasian (Poland)	Healthy subjects	PCR-RFLP	68	100
12	Li et al. [15]	2013	Asian (China)	Healthy subjects	PCR-RFLP	93	83

PCR-RFLP-polymerase chain reaction-restriction fragment length polymorphism, NS-data not shown, DS-direct sequencing.

**Table 2:** Meta-analysis of the association between MTHFR SNP rs1801133 and ONFH risk.

Contrast model	Studies	Odds ratio		Model	*P*_ Heterogeneity_
		OR (95% CI)	*P*_ OR_		
Total studies	12				
T versus C	11	1.15 (0.97, 1.38)	0.095	Random	0.027
TT versus CC	11	1.15 (0.91, 1.46)	0.227	Fixed	0.234
TT/CT versus CC	12	1.09 (0.95, 1.25)	0.222	Fixed	0.245
TT versus CT/CC	11	1.16 (0.93, 1.45)	0.178	Fixed	0.188
Caucasians	7				
T versus C	6	1.12 (0.82, 1.53)	0.428	Random	0.073
TT versus CC	6	0.99 (0.62, 1.61)	0.983	Fixed	0.336
TT/CT versus CC	7	1.13 (0.90, 1.41)	0.295	Fixed	0.208
TT versus CT/CC	6	0.93 (0.59, 1.46)	0.742	Fixed	0.258
Asians	5				
T versus C	5	1.19 (0.93, 1.51)	0.138	Random	0.037
TT versus CC	5	1.21 (0.92, 1.58)	0.164	Fixed	0.141
TT/CT versus CC	5	1.07 (0.90, 1.27)	0.460	Fixed	0.271
TT versus CT/CC	5	1.25 (0.97, 1.60)	0.086	Fixed	0.161

OR: odds ratio, 95% CI: 95% confidence interval, Random: random effects model, Fixed: fixed effects model.
